# Exclusive breastfeeding prenatal intentions among HIV-positive mothers in Blantyre, Malawi: a correlation study

**DOI:** 10.1186/1471-2393-13-203

**Published:** 2013-11-07

**Authors:** Ursula K Kafulafula, Mary K Hutchinson, Susan Gennaro, Sally Guttmacher, Andrew Kumitawa

**Affiliations:** 1 Kamuzu College of Nursing, P.O. Box 415, Blantyre, Malawi; 2 New York University School of Nursing, 726 Broadway, 10th Floor, New York, NY 10003, USA; 3 Boston College, Connell School of Nursing, Boston, MA, USA; 4 College of Medicine, P/Bag 360, Chichiri, Blantyre, Malawi

**Keywords:** Prenatal intentions, Intended duration of exclusive breastfeeding, HIV-positive mothers, Behavioral beliefs, Normative beliefs, Control beliefs

## Abstract

**Background:**

Exclusive breastfeeding is an important component of child survival and prevention of mother-to-child transmission of HIV in resource-poor settings like Malawi. In Malawi, children under the age of six months are exclusively breastfed for an average duration of 3.7 months. This falls short of the recommendations by the World Health Organization as well as the Malawi Ministry of Health that mothers exclusively breastfeed for the first six months of the child’s life. Understanding factors that influence exclusive breastfeeding duration among HIV-positive mothers is important in promoting exclusive breastfeeding among these mothers. An exploratory study was therefore conducted to determine factors that influence HIV-positive mothers’ prenatal intended duration of exclusive breastfeeding and their likelihood to exclusively breastfeed for six months.

**Methods:**

This paper is based on data from a longitudinal, descriptive and correlation study that was conducted at Queen Elizabeth Central Hospital in Blantyre, Malawi between May 12, 2009 and March 22, 2010. Theory of Planned Behavior guided the study. A face-to-face survey was utilized to collect data from a convenience sample of 110 HIV-positive mothers who were at least 36 weeks pregnant at baseline. A modified and pre-tested breastfeeding attrition prediction tool was used to measure exclusive breastfeeding beliefs, intentions and external influences at baseline. Data were analyzed using descriptive and association statistics. Additionally, multiple regressions were run to determine significant predictors of HIV-positive mothers’ prenatal intended duration of exclusive breastfeeding and their likelihood to exclusively breastfeed for six months.

**Results:**

Results revealed high exclusive breastfeeding prenatal intentions among HIV-positive mothers. Prenatal intended duration of exclusive breastfeeding was positively associated with normative, control beliefs and negatively associated with positive beliefs, maternal education and disclosure of HIV status.

**Conclusions:**

Current results suggest that assessment of mothers’ level of education and their positive beliefs towards exclusive breastfeeding may help to identify mothers who are at risk of discontinuing exclusive breastfeeding. Interventions to promote exclusive breastfeeding could include provision of appropriate skills, support and information to help HIV-positive mothers gain control over exclusive breastfeeding.

## Background

Exclusive breastfeeding (EBF) is a better and safer option for feeding infants in most of the sub-Saharan Africa. EBF (defined as feeding an infant no fluids or other feeds other than breast milk for the first six months of the infant’s life) reduces childhood morbidity and mortality from diarrheal diseases [1,2]. In addition, EBF carries a 4-10 fold decreased risk of mother-to-child transmission (MTCT) of HIV compared to mixed feeding during the infant’s first six months of life [3,4]. The World Health Organization [5] and the Malawi Ministry of Health [6,7] recommend that mothers exclusively breastfeed their children for the first six months of the child’s life as part of prevention of mother-to-child transmission (PMTCT) of HIV.

The rates of EBF among HIV-positive mothers in the sub-Saharan Africa [8-10] range from 19% to 48% at four months of life. The current researchers did not cite any study of HIV-positive mothers in Malawi on rates of EBF. However, the 2010 Malawi Demographic and Health Survey estimated the rate of EBF for children less than six months for the general population of mothers to be 71%. Despite this high national (Malawi) rate of EBF [11], studies of mothers in the general population in some parts of Malawi [12-15] found low rates of EBF with the highest rates being 39.1% and 7.5% at two and six months of life respectively. In Vaahtera and colleagues’ [15] study, the EBF rate at three months was very low at 2%. In 2009 (the year the data collection for this study was initiated), the median duration of EBF was 2.5 months and 1.3 months for Malawi and Blantyre district, respectively [16]. Although the median duration of EBF in Malawi has since increased from 2.5 [16] to 3.7 months [11], it still falls short of the WHO’s [5] recommendations and the Malawi national goals [6] for the PMTCT of HIV initiative [7] to have all mothers exclusively breastfeed for six months.

Previous studies in Malawi have identified several factors that contribute to rates of EBF including; socioeconomic factors [15], place of delivery [13], beliefs about milk sufficiency and demands on the mother’s body [14,17], and pre-lacteal feeding practices [13-15]. Only one of these studies [17] included HIV-positive women and none were conducted in Blantyre.

One of the major challenges facing Malawi and other sub-Saharan African countries that are severely affected by HIV and AIDS is to develop effective interventions to promote exclusive breastfeeding. However, health promotion programs are more likely to be effective if they are theory based, culture-specific, and tailored to the target population [18,19]. Little is known about the factors that influence exclusive breastfeeding among HIV-positive mothers in Blantyre, Malawi hence the need to explore factors that influence prenatal intended duration of exclusive breastfeeding among HIV-positive mothers in Blantyre, Malawi.

### Theoretical framework

The Theory of Planned Behavior (TPB) [20] guided this study. TPB provides a useful framework for predicting and understanding social and health behavior [21]. The main concepts of this theory are behavior, intention, behavioral attitude, subjective norm and perceived behavioral control. The theory posits that a person’s intention to perform a behavior is the principle predictor of behavior. However, behaviors over which an individual has limited control can be predicted with considerable accuracy from intentions and an individual’s perceptions of behavioral control [20]. Behavioral attitude, subjective norm and perceived behavioral control are the determinants of intention, which in turn are determined by their corresponding behavioral, normative and control beliefs. These corresponding beliefs are influenced by factors external to the individual such as socioeconomic status.

According to Ajzen [20], behavioral intention is the motivation or desire for someone to engage or not to engage in a behavior. An attitude toward a behavior is a psychological tendency of favoring or disfavoring something, and is influenced by a person’s beliefs about the consequences of performing a particular behavior (behavioral beliefs). Subjective norms are a person’s judgments about significant others’ preferences concerning a particular behavior. One’s perception of whether people who are important in his/her life would approve or disapprove of a behavior (normative belief) affects one’s subjective norms for that particular behavior [20]. Perceived behavioral control is a person’s perception about how easy or difficult it is to perform a behavior, and it is influenced by the individual’s beliefs about whether he or she possesses appropriate skills and resources to perform the behavior (control beliefs). Perceived behavioral control takes into account two sets of factors that an individual has to consider in determining his/her capabilities to perform a behavior. These factors are called internal factors (abilities, skills, compulsions) or external factors (time availability, opportunity, resources) [20,21].

**Figure 1:**
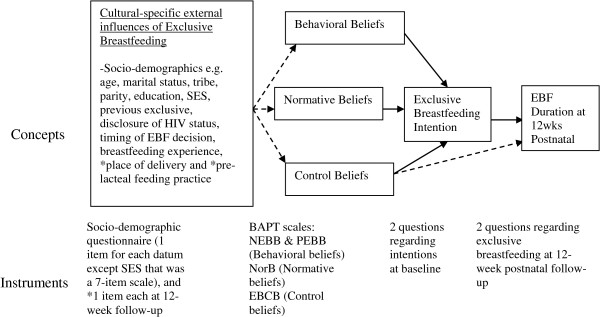
Conceptualization of Theory of Planned Behavior in the present study.

The assessment basis for behavioral attitudes, subjective norms and perceived behavioral control is the individual’s evaluation of the corresponding accessible beliefs [20]. In this study, the accessible beliefs (behavioral, normative and control) were used to represent behavioral attitudes, subjective norms, and perceived behavioral control. TPB was chosen for this study because besides considering the individual’s cognitive processes, it also takes into consideration factors external to the individual which include significant others. Figure 1 illustrates how TPB was conceptualized in this study.

## Methods

### Design

The design of the study on which this article is based was longitudinal, descriptive and correlation. The study was part of a large project that aimed at exploring culture-specific influences of exclusive breastfeeding among HIV-positive mothers in Blantyre, Malawi. The large project employed both quantitative and qualitative methods. This paper is based on the baseline data from the quantitative phase of this project.

### Study setting

This study was conducted at Queen Elizabeth Central Hospital (QECH) maternity unit in Blantyre, Malawi. Blantyre is one of the districts in the Southern Region of Malawi. It has a total population of 1.01 million, 68% of whom live within the urban area [22]. The district is served by QECH, a public hospital which functions both as a district hospital for Blantyre and as a referral hospital for the southern region of Malawi. QECH is also a teaching hospital for different health related professions. Blantyre is one of the districts in Malawi with high prenatal care coverage. Most of the women in Blantyre receive prenatal care from skilled health care providers with 92.2% of them cared for by midwives. Seventy percent of women in Blantyre deliver in public health facilities [16]. QECH has organized services for PMTCT of HIV and prenatal HIV testing is an important gateway into these services. Availability of PMTCT services provided access for research to HIV-positive prenatal and postnatal mothers without asking them to go through HIV screening in order to participate in the study.

### Participants

The study utilized a convenience sample of 110 HIV-positive pregnant women. To participate in this study, one had to be at least 18 years old and with a singleton pregnancy, at least 36 weeks pregnant, intending to breastfeed her baby (whether exclusively or not), and a resident of Blantyre district. Furthermore, one had to be able to hear, understand and speak Chichewa (a national language for Malawi), willing to give informed consent, and be re-interviewed at 12 weeks postnatal. Women who were experiencing tuberculosis (TB) or full-blown AIDS (self reported record of TB or a record of being chronically sick) were excluded from this study.

### Sample size and power calculation

A minimum sample size of 87 was estimated by GPower 3.0.8 for multiple linear regression analysis using 13 variables: 3 belief constructs of TPB measured by 4 subscales of the Breastfeeding Attrition Prediction Tool (BAPT), and 9 socio-demographic variables (external influences) at alpha value of 0.05, power of 0.80, and effect size of 0.24. The effect size of 0.24 represented the smallest effect size from literature review of studies on breastfeeding from other, mostly industrialized countries that had used the Theory of Planned Behavior [23-25]. One hundred and ten (110) participants were recruited in order to allow for an attrition rate of 23%.

### Ethical consideration

New York University Committee on Activities Involving Human Subjects and Malawi College of Medicine Research and Ethics Committee approved this study. In addition, the researchers sought permission from the director of Queen Elizabeth Central Hospital (QECH), Head of Obstetric and Gynecology Department and Head of Pediatric Department of QECH to access participants. Written informed consent was obtained from individual participants.

### Data collection

The researchers collected data between May 12, 2009 and March 22, 2010 utilizing a structured and modified exclusive breastfeeding attrition prediction tool. Care providers of participants were not involved in data collection.

### Instrumentation and measures

Beliefs (behavioral, normative and control) were measured at interval level utilizing the four sub-scales of the exclusive breastfeeding attrition prediction tool (EBAPT). EBAPT was adapted from the original breastfeeding attrition prediction tool (BAPT) [26].

### Behavioral beliefs

Negative exclusive breastfeeding beliefs (NEBB) sub-scale (12 items) and positive exclusive breastfeeding beliefs (PEBB) sub-scale (13 items) measured behavioral beliefs. Each item was a weighted item, which was obtained by multiplying each behavioral belief by its corresponding motivation to comply statement. Items included: (1) ‘*Exclusive breastfeeding is more convenient than mixed feeding’; ‘Using a feeding method that is convenient is…’* (2) *‘It is difficult to exclusively breastfeed’; ‘Using a feeding method that is easy to do is…’.* Response choices for each belief and its corresponding motivation to comply were on a 5-point Likert scale ordered on a continuum from 1 = strongly disagree to 5 = strongly agree; and 1 = not important at all to me to 5 = very important to me; respectively. Addition of all the weighted items in the sub-scale produced a total score for the sub-scale. Total possible scores ranged from 12-300 and 13-325 for NEBB and PEBB respectively. The higher the score of NEBB, the stronger the negative exclusive breastfeeding beliefs the HIV-positive woman had. Likewise, the higher the score of PEBB, the stronger the positive exclusive breastfeeding beliefs the HIV-positive woman had.

### Normative beliefs

Normative belief (NorB) sub-scale (10 items) measured normative beliefs. As for NEBB and PEBB sub-scales, each item was a weighted item obtained by multiplying each normative belief by its corresponding motivation to comply statement. Items and their corresponding motivation to comply included: (1) *‘The baby's father thinks I should…’; ‘How much do you care about the baby’s father’s opinion on how you should feed your baby?’;* (2) *‘My nurse-midwife thinks I should…’; ‘How much do you care about your nurse-midwife’s opinion on how you should feed your baby?’* Response choices for each normative belief and its corresponding motivation to comply were ordered from 1 = definitely not exclusively breastfeed to 5 = definitely exclusively breastfeed; and 1 = I do not care at all to 5 = I care very much; respectively. The response choices also provided participants with an option of 0 = not Applicable. A total score for the sub-scale was obtained by adding all the weighted scores. Total possible scores ranged from < 10-250. The higher the score, the greater the support the HIV-positive woman had for exclusive breastfeeding.

### Control beliefs

Exclusive breastfeeding control beliefs (EBCB) sub-scale (10 non-weighted items) measured control beliefs. Items included: *‘I have the necessary skills to exclusively breastfeed,’ ‘I am determined to exclusively breastfeed’* and *‘I am confident I can exclusively breastfeed’.* Response choices were on a continuum from 1 = strongly disagree to 5 = strongly agree. Total possible scores ranged from10-50. The higher the score, the greater the perceived control the HIV-positive woman had over exclusive breastfeeding. (See the more detailed description of scoring the beliefs’ items of the breastfeeding attrition prediction tool in the Additional file 1).

### External salient culture-specific influences

According to TPB, any factors that may have an effect on behavior do so by influencing behavioral, normative and control beliefs. Such factors are called external influences. Literature review and analysis of data from the qualitative component of the larger study from which this paper comes revealed some socio-demographic factors that may influence exclusive breastfeeding intentions and duration. These factors were socio-economic status (SES), maternal age, marital status, maternal education, previous experience of exclusive breastfeeding, place of delivery, pre-lacteal feeding practices, disclosure of HIV status, and timing of decision to breastfeed the baby. In the current study, these factors are referred to as external salient culture-specific influences. These factors were measured using the demographic items on the EBAPT.

### Intentions to exclusively breastfeed

Two items that are part of EBAPT measured intentions prenatally. The item ‘*How many weeks do you intend to exclusively breastfeed your infant?’* was used to measure intentions at a ratio level. Because, at the time of this study, all prenatal HIV-positive women who could not afford infant formula in Malawi were advised to exclusively breastfeed their infants for the first six months [6], measuring prenatal exclusive breastfeeding intentions on a Yes/No was likely to produce less reliable results due to social desirability [27]. Therefore, the researchers decided to measure intentions as intended duration of exclusive breastfeeding in weeks to reduce the social desirability effect. However, since intentions are often measured on a Likert scale [20], a second item ‘*How likely is it that you will exclusively breastfeed your baby for six months?’* was also used to measure intentions. The item was on a 5-point Likert scale with 5 = very likely; 4 = likely; 3 = not sure; 2 = unlikely; 1 = very unlikely.

### Actual exclusive breastfeeding duration in weeks

The actual duration of exclusive breastfeeding in weeks was assessed at 12 weeks postnatal follow-up. We chose a 12-week follow-up point because it was beyond both national (Malawi) and local (Blantyre district) median durations of exclusive breastfeeding; 10 weeks (2.5 months) and approximately 5 weeks (1.3 months) respectively [16]. Therefore, a follow-up at 12-weeks postnatal allowed the researchers to identify factors associated with mothers who were still exclusively breastfeeding their infants. If the postnatal follow-up was later than 12 weeks, for example at 24 weeks postnatal, few if any mothers would still be exclusively breastfeeding.

A 7-item questionnaire was used. The items included: (1) the birth date of the infant and (2) feeds that were given to the infant before lactation was established. This helped the researchers to determine initiation of exclusive breastfeeding; (3) feeds other than breast milk that were being given to the infant at the time of the follow-up helped the researchers to confirm exclusive breastfeeding for those who indicated that they were still exclusively breastfeeding their infants; and (4) age of baby in weeks when feeds other than breast milk were initiated to determine time when exclusive breastfeeding was terminated.

### Reliability of the instrument

The original BAPT [26] has an overall reliability of 0.80, with negative breastfeeding sentiments (NBS) having a Chronbach alpha value of 0.83, positive breastfeeding sentiments (PBS) 0.79, social and professional support sub-scale (SPS) 0.85, and breastfeeding control (BFC) 0.81. The item loading for each sub-scale was: NBS 0.31-0.70, PBS 0.30-0.60, SPS 0.31-0.75, and BFC 0.41-0.76. In addition, the original BAPT has a predictive validity of 73% [26].

BAPT has been tested with both prenatal [28,29] and postnatal [28,30] women, and has demonstrated adequate reliability ranging from 0.77-0.93 for all the sub-scales in most studies. In the current study, estimates of internal consistency using Cronbach’s Alpha coefficient were computed for each sub-scale in the EBAPT instrument and EBAPT as a whole using the Statistical Package for Social Science (SPSS). Results revealed a good internal consistency in all the four sub-scales of the BAPT and BAPT as a whole with Cronbach’s alpha of greater than .80.

The 12-item NEBB, 13-item PEBB and 10-item EBCB sub-scales had a Cronbach’s alpha of .881, .893 and .917 respectively. There was no item with a corrected item-Total correlation (Item loading) of less than .3 in all of these sub-scales. This indicates that all the items in these sub-scales were measuring the same thing [31]. The 10-item NorB sub-scale had an alpha of .846. There was one item ‘*My Traditional Birth Attendant thinks I should EBF’* with a corrected item-Total correlation of less than .3. However, the suggested Cronbach’s alpha if this item was deleted was .856, which did not differ greatly from the overall Cronbach’s alpha of .846 for the sub-scale. Therefore, the item was retained in the sub-scale. Finally, the 45-item EBAPT instrument as a whole had a Cronbach’s alpha of .858. There were five items with a corrected item-Total correlation of less than .3. An evaluation of the suggested Cronbach’s alpha values if each of these items were deleted one at a time did not greatly increase the overall alpha value for the EBAPT instrument. These suggested alpha values ranged from .852-.862 compared to the overall alpha value of .858. This indicates that the 45-item BAPT instrument was a reliable instrument for measuring exclusive breastfeeding beliefs.

### Data analysis

Data analysis was done using SPSS version 16.0 for windows. Pearson’s product-moment coefficient, Spearman’s rho coefficient and Pearson’s Chi-square test for independence were used to explore associations among the variables. To be consistent with previous studies that have used Janke’s original BAPT instrument and TPB, multiple linear regression analyses were run with number of weeks of intended duration of exclusive breastfeeding at baseline. A sequential modeling approach was used. All the external salient culture-specific influences were entered together at the first step, and then behavioral, normative and control beliefs, one at a time, to assess their effects on the outcome variables. Only the predictor variables that were significantly associated with prenatal intended duration of EBF were entered into the models. However, because some variables did not meet the assumption of normalcy, logistic regression analyses were also run to confirm the findings.

## Results

### Demographic data

Table 1 presents details of the participants’ demographic information. Based on a socio-economic status (SES) 7-item scale, with minimum and possible highest scores of 2 and 9 respectively, the majority (83%) of the participants were classified as middle SES (score of 5-8).

**Table 1:** Characteristics of participants at baseline (n = 110)

**Characteristic**	**Frequency (%)**	**Characteristic**	**Frequency (%)**
**Maternal education**		**Tribe**	
None	1(0.9)	Ngoni	29(26.4)
Primary school	39(35.5)	Lomwe	27(24.5)
Junior Certificate	19(17.3)	Yao	17(17.3)
MSCE	36(32.7)	Chewa	13(11.8)
College	15(13.6)	Sena	7(6.4)
		Tumbuka	6(5.5)
		Mang’anja	4(3.6)
		Tonga	3(2.7)
		Khokhola	1(0.9)
		mchangani	1(0.9)
**Marital status**			
Married	101(91.8)	**Previous EBF experience**	
Divorced	4(3.6)	Yes	87(78.4)
Co-habiting	2(1.8)	No	13(21.6)
Widow	2(1.8)		
Never married	1(0.9)	**Disclosure of HIV status to Spouse or/and parent**	
		Yes	70(63.6)
**Parity**		No	40(36.4)
Nulliparous	20(18)		
Low parity (1-3)	77(70)	**Timing of decision to breastfeed**	
High parity (4-8)	13(12)	Before conception	5(4.5)
		During 1^st^ trimester	29(26.4)
**Socio-economic status**		During 2^nd^ trimester	61(55.5)
Low (score of 2-4)	10(9.1)	During 3^rd^ trimester	15(13.6)
Medium (score of 5-8)	91(82.7)		
High (score of 9-12)	9(8.2)		

### Behavioral beliefs

The participants’ mean scores for the individual negative exclusive breastfeeding beliefs (NEBB) items ranged from 8.93 (SD = 3.694) to 12.98 (SD = 4.842) with a possible maximum score of 25 for each item. These mean scores indicated that the participants had low to moderately high negative exclusive breastfeeding beliefs. Mean scores for the individual positive exclusive breastfeeding beliefs (PEBB) items ranged from 12.25 (SD = 6.057) to 18.82 (SD = 3.528). Except for one item (*Mixed breastfed babies are more fussy*; mean = 12.25), all the PEBB item mean scores were greater than 15 indicating that the participants had high positive exclusive breastfeeding beliefs. See Table 2 for details of the summed up weighted scores for both NEBB and PEBB sub-scales.

**Table 2:** Description of EBAPT tool and participants’ scores on beliefs sub-scales at baseline (n = 110)

**EBAPT sub-scale**	**Number of items in each sub-scale**	**Total possible scores in each sub-scale**	**Participants’ scores in each sub-scale**
Behavioral Beliefs			
NEBB	12	12-300	56-258, median =132, IQR = 53
PEBB	13	13-325	148-320, median = 210, IQR = 54
Normative Beliefs			
(NorB)	10	0-250	0-225, median = 65, IQR = 70
Control Beliefs (EBCB)	10	10-50	19-50, mean =40.15, SD = 6.743
Complete EBAPT Tool	45	— — — —	— — — —

Note: EBAPT = exclusive breastfeeding attrition prediction tool; NEBB = negative exclusive breastfeeding belief; PEBB = positive exclusive breastfeeding belief; NorB = normative belief; EBCB = exclusive breastfeeding control belief.

### Normative beliefs

The participants’ mean scores for the individual normative beliefs (NorB) items ranged from 0.25(SD = 2.047) to 16.12(SD = 6.103). The lowest scores were on items asking about expectations of traditional birth attendants and other people important in the mother’s life regarding EBF. Highest mean scores were on items about expectations of nurse-midwives (Mean = 16.12; SD = 6.103), the father of the baby (Mean = 14.41; SD = 8.628) and their doctor (Mean =13.54; SD = 8.057) regarding EBF. The summed up total NorB scores are displayed in Table 2.

### Control beliefs

The participants’ mean scores for the individual control beliefs (EBCB) items ranged from 3.60 (SD = 1.151) to 4.20 (SD = .764). Except for one item ‘*I won’t need help to EBF* ’ , participants scored more than 75% of the possible maximum scores indicating high exclusive breastfeeding control beliefs. The participants’ actual total EBCB scores are displayed in Table 2.

### Prenatal intentions

Seventy seven percent of participants reported that they were likely to exclusively breastfeed, 11.8% were not sure while 10.9% were unlikely going to exclusively breastfeed their babies for 24 weeks. The prenatal intended duration of exclusive breastfeeding ranged from 4-24 weeks. Mothers intended to EBF their infants for different reasons. These reasons included lack of money to purchase infant formula, belief that breast milk is the best, and to conceal their HIV status because choosing not to breastfeed was seen as self-revealing one’s HIV status.

### Correlates of prenatal intentions of exclusive breastfeeding

Prenatal intended duration of EBF was significantly associated with maternal education (r = -.417, p < .001), parity (r = .213, p < .05), disclosure of HIV status (r = -.195, p < .05), PEBB (rho = -.198, p < .05), NorB (r = .194, p < .05) and EBCB (r = .286, p < .01). The likelihood of HIV-positive mothers to exclusively breastfeeding for six months was significantly associated with NorB [X^2^ = 8.553, phi = .279, p < .01], and EBCB [X^2^ = 16.589, phi = .388, p < .001).

The prenatal intended duration of EBF was then dichotomized as “Less than 24 weeks and Equal to 24 weeks’ and its relationship with the rest of the variables was re-assessed through Pearson’s chi-square test to compare the results with those obtained through Pearson’s product-moment correlation coefficient as a way of validating them. Statistically significant associations were observed between: (1) prenatal intended duration of EBF and maternal education [X^2^ = 21.686, phi = -.444, p < .01], PEBB [X^2^ = 4.089, phi = -.193, p < .05], NorB [X^2^ = 3.945, phi = .189, p < .05], and EBCB [X^2^ = 4.980, phi = .213, p < .05]. Although non-parametric analyses failed to demonstrate significant association between prenatal intended duration and parity (Fisher’s Exact Test at p = .09, phi = .169, p > .05) and disclosure of HIV status (Fisher’s Exact Test at p = .079, phi = -.168, p > .05), the direction of the associations and the rest of the findings were consistent with those obtained from parametric analyses.

Prenatal intentions were positively associated with EBF at 12 weeks postnatal (r = .106; rho = .251). However, the relationship was not statistically significant (p > .05). This may be due to the reduced statistical power of the sample at 12 weeks postnatal because of the high loss to follow up that this study suffered. Fifty-five out of 110 participants returned for follow up at 12 weeks postnatal. Analysis of attrition bias was conducted. The ‘*drop-outs*’ and the ‘*non-drop-outs*’ were not significantly different on all the baseline variables except for two (prenatal intentions and normative beliefs scores) variables. At baseline, 83.6% of the participants who came back for their 12 weeks postnatal follow-up reported that they intended to EBF their babies for six months compared to 70.9% of those who dropped out of the study [X^2^ = 11.217 (2, 55); Cramer’s value = .319; p = .004]. Furthermore, Mann Whitney U Test revealed a significant difference in the total NorB scores of those who returned for the 12 week follow up (Median = 80, n = 55) and those who did not come for the 12 week follow up (Median = 60, n = 55), U = 1190, z = -1.929, p = 0.05, r = -.184. Considering the significantly high prenatal intentions for EBF and high NorB scores indicating a high prenatal motivation for EBF and a high possible support for EBF from significant others respectively, the ‘*non-drop outs*’ group had high opportunity for EBF for longer periods. Social desirability may not fully explain these findings.

### Predictors of prenatal intended duration of exclusive breastfeeding

Multiple linear regression analyses were run with number of weeks of intended EBF duration as an outcome variable. A sequential modeling approach was used. Maternal education, parity and disclosure of HIV status were entered together at the first step, and then behavioral beliefs (PEBB) at second step, normative beliefs (NorB) at third step and control beliefs (EBCB) at fourth step to assess their effects on number of weeks of intended EBF. Table 3 presents the results from multiple linear regressions. The model containing maternal education, parity, disclosure of HIV status, PEBB, NorB and EBCB explained about 35% of the variance in number of weeks of intended duration of EBF. In the order of their strength, starting with the strongest, EBCB, maternal education and PEBB were significant predictors of prenatal intended duration of EBF. Although addition of PEBB to the model did not significantly improve R^2^, it became a significant predictor (p < .01) of prenatal intended duration of EBF after EBCB was added in the final model. NorB, that significantly improved R^2^ when added to the model, was not a significant predictor of prenatal intended duration of EBF when EBCB was added to the equation. This suggests some possible mediation effect in the relationships. However, this was not explored further because it was beyond the realm of the current study.

**Table 3:** Multiple linear regression predicting intended duration of EBF in weeks at baseline (n = 110 across all models)

**Predictor**	**Model 1**	**Model 2**	**Model 3**	**Model 4**
Constant	26.596(2.747)	30.152(15.570)	33.285(15.308)	61.246(15.737)
	***		*	***
Maternal Education	-2.295(.581)	-2.272(.592)	-2.262(.580)	-2.154(.540)
	-.390***	-.386***	-.385***	-.366***
Parity	.221(.962)	.223(.966)	.259 (.946)	-.045(.883)
	.023	.023	.027	-.005
Disclosure of HIV status to Spouse & family	-1.693(.967)	-1.669(.977)	-1.573(.957)	-1.113(.897)
	-.153	-.151	-.142	-.101
Positive Beliefs (PEBB)		-1.544(6.654)	-3.180(6.554)	-20.610(7.394)
		-.021	-.043	-.278**
Normative Beliefs (NorB)			2.631(1.126)	1.700(1.071)
			.201*	.130
Control Beliefs (EBCB)				.328(.079)
				.415***
R^2^	.197	.198	.238	.348
R^2^ change	.197***	.00	.040*	.110***
Adjusted R^2^	.175	.167	.201	.310
Model pvalue	P < .001	P < .001	P < .001	P < .001

Note: PEBB = positive exclusive breastfeeding belief; NorB = normative belief; EBCB = exclusive breastfeeding control belief.

Cell entries are estimated unstandardized coefficients (std error) and standardized coefficients; *p < 0.05, **p < 0.01, ***p < .001.

Logistic regression analyses were also run to predict prenatal intended duration of EBF to validate the findings. For this, prenatal intended duration was categorized into two (0 = Less than 24 weeks of EBF; 1 = Equal to 24 weeks of EBF). Block 1 and the model as a whole containing maternal education as a predictor, was statistically significant (X^2^ = 23.141, p < .001). Addition of: (1) PEBB in Block 2 resulted in no significant contribution (X^2^ = .866, p = .352) to the model; (2) NorB in Block 3 resulted in a significant contribution (X^2^ = 5.409, p < .05) to the model; and (3) EBCB contributed significantly to the model [X^2^ = 4.947, p < .05). The final model containing all the predictors was statistically significant, X^2^ (4, n = 110) = 34.363, p < .001 indicating that the model was able to distinguish between HIV-positive mothers who reported and those who did not report that they would EBF for 24 weeks. The chi-square value for the Hosmer-Lemeshow Goodness of Fit Test was 5.850 with a significance level of p = .440; indicating support for the model. The model as a whole explained 26.8% (Cox and Snell R square) and 39.9% (Nagelkerke R square) of the variance in prenatal intended duration of EBF in weeks, and overall correctly classified 78.2% of HIV-positive mothers. The Wald statistics showed that only maternal education (p < .001) and EBCB (p < .05) made a significant contribution to the final model predicting prenatal intended duration of EBF. See Table 4.

**Table 4:** Logistic regression analysis predicting intended duration of EBF in weeks at baseline (n = 110)

**Predictor**	**B**	**S.E.**	**Wald**	**df**	**Sig.**	**Exp(B)**	**95% C.I. for Exp(B)**	
							**Lower**	**Upper**
Constant	1.945	.586	10.996	1	.001	6.991	-	-
Maternal education	-2.395	.626	14.614	1	.000	.091	.027	.311
PEBB	-1.109	.617	3.233	1	.072	.330	.099	1.105
NorB	1.405	.861	2.664	1	.103	4.075	.754	22.013
EBCB	1.330	.620	4.610	1	.032	3.782	1.123	12.740

Note: PEBB = positive exclusive breastfeeding belief; NorB = normative belief; EBCB = exclusive breastfeeding control belief.

Although PEBB was a significant predictor of prenatal intended duration of EBF when data were analyzed through multiple linear regression, it had a borderline significance (Wald = 3.233, p = .072; OR = .330, 95% CI; .099 to 1.105) when logistic regression was used. The strongest predictor of prenatal intended duration was EBCB with an odds ratio (OR) of 3.782, CI; 1.123-12.740; Wald = 4.610, p < .05 indicating that HIV-positive mothers with high EBCB scores were about 4 times more likely to intend to EBF for 24 weeks compared to those who had low EBCB scores. Higher maternal education had OR of .091, 95% CI; .027 to .311 indicating that HIV-positive mothers with higher education (≥MSCE) were .909 (91%) less likely to intend to exclusively breastfeed for 24 weeks than those who had less than MSCE qualification.

Results from multiple linear regression analyses have demonstrated that maternal education, PEBB, and EBCB were significant predictors of prenatal intended duration of EBF. These results were consistent with those obtained from multiple logistic regression except that PEBB failed to reach significant level (p = .072).

### Predictors of likelihood of HIV-positive mothers to EBF for 24 weeks

Sequential logistic regression analysis was used to predict likelihood of HIV-positive mothers to EBF for 24 weeks (assessed as 0 = unlikely and not sure; 1 = likely) with normative beliefs (NorB) and exclusive breastfeeding control beliefs (EBCB) as predictors. These two variables were the only possible predictors that showed a statistically significant association with likelihood of EBF for 24 weeks. The model containing NorB and EBCB as predictors of likelihood of EBF for 24 weeks was statistically significant, X^2^ = 23.528, p < .01. Hosmer and Lemeshow Test of Goodness of fit at significance level of P > .05. The model explained 19.3% (Cox and Snell R square) and 29.3% (Nagelkerke R square) of the variance in the likelihood of HIV-positive mothers to EBF for 24 weeks, and overall correctly classified 77.3% of the HIV-positive mothers. Only EBCB (Wald = 9.421, p < .01) made a significant contribution to the model predicting the likelihood of EBF for 24 weeks. The OR was 4.706 with 95% CI of 1.750 to 12.652 indicating that HIV-positive mothers with high EBCB scores were almost 5 times more likely to EBF for 24 weeks than their counterparts with lower EBCB scores.

## Discussion

Results from this study have revealed high EBF prenatal intentions among the HIV-positive mothers. This is in line with the results of studies of HIV-positive mothers conducted in India [32] and Nigeria [33]. However, the current results contradict those of a study in South Africa [34] and in Cote d’Ivoire [35] that found low EBF intentions among HIV-positive mothers. A possible explanation for this difference is that HIV-positive mothers in the South Africa and the Cote d’Ivoire studies had an alternative to breast milk because those who did not choose to breastfeed their babies were given free infant formula. At QECH and Malawi as a whole, HIV-positive mothers are not given free infant formula. This was also true with the participants in the India and Nigeria studies whose results were consistent with those of the current study.

Based on TPB [20], the participants in the present study were likely to exclusively breastfeed their babies for long duration since breastfeeding intentions are the principle predictors of breastfeeding behaviors. Although the results of the current study were not statistically significant, probably due to the reduced statistical power of the sample, prenatal intentions were positively associated with actual EBF duration at 12 weeks postnatal. Therefore, strengthening influences of these participants’ EBF intentions such as normative (support) and control beliefs related to EBF may promote duration of EBF among the participants.

### Correlates of prenatal intentions

In the present study, HIV-positive mothers who had given birth before had higher prenatal intentions for EBF than those who were expecting their first babies. Consistent with TPB [20], mothers who perceived more EBF support from their significant others and those who felt that they had more control over EBF respectively, intended to exclusively breastfeed for longer duration and were more likely to EBF for 24 weeks than those who did not. Contrary to what TPB postulates, high scores of positive exclusive breastfeeding beliefs (PEBB) predicted reduced prenatal intended duration of EBF. According to TPB [20], one would expect a high score of positive beliefs to predict an increased prenatal intended duration of EBF. This was not the case in the current study. It is possible that in this sample, a complex relationship existed between PEBB and prenatal intended duration of EBF leading to results inconsistent to TPB. The mothers’ fear of exposing their babies to HIV virus, which emerged as an important reason for discontinuing EBF before 12 weeks postnatal, might be a possible extraneous variable that influenced the relationship between PEBB and prenatal intended duration of EBF. The independent influence of the mothers’ positive exclusive breastfeeding beliefs on their prenatal intentions may have been over-shadowed by their fear of passing the HIV onto their babies. However, further research is needed to explore this relationship.

In contrast to results from a previous study [31], higher maternal education in the current study was associated with reduced prenatal intended duration of EBF. HIV-positive mothers who had an academic qualification of equal or more than MSCE intended to EBF for a shorter period than their counterparts with a qualification of less than MSCE. This finding could be because mothers who had higher academic qualifications were likely to be financially empowered and could therefore afford to pay for infant formula, a better alternative for feeding their babies in the presence of maternal HIV infection. These mothers were therefore, less likely to intend to EBF their babies for a long duration because they could afford to replace breast milk with infant formula once they stopped breastfeeding. Additionally, mothers who intended to breastfeed for a short period and had a qualification of MSCE or more, may have been among those who intended to breastfeed because they wanted to conceal their HIV status from other people rather than because of lack of money for purchasing infant formula. However, no exploration to this effect was done in the present study.

HIV-positive mothers who had disclosed their HIV status to their spouses and/or family members intended to EBF for a short duration. This is in contrast with the anticipated benefits of HIV disclosure for optimal uptake and adherence to PMTCT interventions. EBF is one of the PMTCT interventions. At the time this study was being conducted, EBF was the recommended option for mothers who did not satisfy the AFASS (affordable, feasible, accessible, sustainable and safety) criteria for replacement feeding. In the present study, it is possible that mothers who had disclosed their status feared opposition from their significant others because of the perceived and actual risks of HIV transmission through breast milk hence the shorter prenatal intended duration of EBF than their counterparts. Since no previous literature was cited for comparison, these findings necessitate the need for more research to further explore the relationship between prenatal intentions and disclosure of HIV status among HIV-positive mothers.

### Strengths and limitations of the study

The use of multiple analytical methods helped to validate the findings thereby building a strong basis for possible intervention studies. However, significant limitations also exist. There is limited generalization of findings from this study because of the use of a convenience sample. Although the findings may be applicable to other HIV-positive mothers in Blantyre, generalization of these findings to HIV-positive mothers of different socio-economic status may be limited because participants were drawn from one site that caters mainly for low-income women. It is not known what the EBF prenatal intentions of HIV-positive mothers of high SES would be. Further research is therefore, needed to better understand factors that influence EBF in HIV-positive mothers.

## Conclusions

The aim of the current study was to explore factors that influence HIV-positive mothers’ prenatal intended duration of exclusive breastfeeding and their likelihood to exclusively breastfeed for 24 weeks in Blantyre, Malawi. Face-to-face interviews using a structured EBAPT tool were conducted. Besides confirming results from previous studies from other countries, the results have generated knowledge on culture-specific influences of prenatal intentions among HIV-positive mothers in Blantyre, Malawi. EBF prenatal intentions were associated with several factors, which were maternal education, parity, previous experience of EBF, previous EBF success, disclosure of HIV status to spouse and/or parents, and EBF beliefs.

Despite some inconsistent results in the current study, Theory of Planned Behavior (TPB) may be an important guide in the promotion of EBF in HIV-positive mothers in Blantyre, Malawi. The TPB main concepts of control and normative beliefs were particularly important in predicting prenatal intended duration of EBF. However, positive beliefs and maternal education predicted undesirable outcome, that is, reduced prenatal intended duration of EBF. External influences such as socioeconomic status, marital status and tribal affiliation were not significant determinants of prenatal intended duration of EBF.

Based on these results, maternal positive EBF beliefs and education can be used to identify HIV-positive mothers who may be at risk of early cessation of EBF. Once identified, these mothers may be offered timely and appropriate supportive interventions to promote EBF. The interventions should aim at strengthening the normative and control beliefs of these mothers. However, interventions targeting HIV-positive mothers alone may not be adequate and effective as several other factors beyond these mothers’ control, such as disclosure of one’s HIV status to significant others, may also influence these mothers’ intentions towards EBF.

One-to-one support provided by each HIV-positive mother’s preferable support person may be more appropriate and effective than group support [36-40] and should be encouraged. To ensure and promote this, each HIV-positive pregnant woman should be encouraged to identify a person of her choice during her initial prenatal visit. This person should act as a support system for the HIV-positive woman’s entire pregnancy and postnatal period. However, considering the fact that 84% of the total population of Malawi lives in rural area and that 27% of mothers still deliver outside of a health facility [16], utilizing family members or friends in EBF promotion interventions in Malawi may be more practical and could reach more mothers than utilizing hospital personnel or community trained breastfeeding supporters. Furthermore, such interventions may be more sustainable in a resource-constrained country such as Malawi because no payment for the support system would be required.

## Abbreviations

BAPT: Breastfeeding attrition prediction tool; EBAPT: Exclusive breastfeeding attrition prediction tool; EBCB: Exclusive breastfeeding control beliefs; EBF: Exclusive breastfeeding; FG2P: Focus group two participant; MSCE: Malawi school certificate of education; NEBB: Negative exclusive breastfeeding beliefs; NorB: Normative beliefs; PEBB: Positive exclusive breastfeeding beliefs; PMTCT: Prevention of mother-to-child transmission; QECH: Queen Elizabeth Central Hospital; SES: Socio-economic status; TBA: Traditional birth attendant; TPB: Theory of Planned Behaviour.

## Competing interests

The authors declare that they have no competing interests.

## Authors’ contributions

UKK conceptualized this study, facilitated data collection, and led the data analysis under the supervision of the statistician AK. MKH, SG, and SG advised the senior author on the conceptualization of the study, data collection and presentation of findings. They also critically edited the text. All authors read and approved the final manuscript.

## Pre-publication history

The pre-publication history for this paper can be accessed here:

http://www.biomedcentral.com/1471-2393/13/203/prepub

## Supplementary Material

Additional file 1 **Scoring of exclusive breastfeeding attrition prediction tool (EBAPT).** Describes the scoring of the four subscales of exclusive breastfeeding attrition prediction tool. Click here for file
