# Supplementary material for: Exclusive breastfeeding prenatal intentions among HIV-positive mothers in Blantyre, Malawi: a correlation study
Source: BMC Pregnancy Childbirth. 2013 Nov 7;13:203. doi: 10.1186/1471-2393-13-203 (PMC3826508; doi:10.1186/1471-2393-13-203)
Supplement: Additional file 1 — Scoring of exclusive breastfeeding attrition prediction tool (EBAPT). Describes the scoring of the four subscales of exclusive breastfeeding attrition prediction tool. [file 1471-2393-13-203-S1.doc]

**Scoring Guidelines for exclusive breastfeeding attrition prediction tool (EBAPT).**

Behavioral Beliefs

- 1. Negative Exclusive Breastfeeding Beliefs (NEBB) Scale
     1. Multiply each belief score by its corresponding outcome evaluation. The items to be multiplied are as follows: 2,37; 3,38; 6,41; 7,42; 10,45; 13,48; 14,49; 16,51; 17,52; 19,54; 20,55; 24,59.

For example: a person scores a 5 for item 2 “exclusive breastfeeding is painful” and a 4 for item 37 "using a feeding method that doesn't cause me pain is ...". These scores are multiplied for an item attitudinal score of 20.

- - 1. Sum all multiplied scores for the Negative exclusive Breastfeeding Beliefs score.
    2. The higher the score, the greater the negative exclusive breastfeeding beliefs.
  1. Positive Exclusive Breastfeeding Beliefs (PEBB) Scale

1. Multiply each belief score by its corresponding outcome evaluation. The items to be multiplied are as follows: 1,36; 4,39; 5,40; 8,43; 9,44; 11,46; 12,47; 15,50; 18,53; 21,56;22,57; 23,58; 25,60.

For example: a person scores a 2 for item 5 "Breastmilk is healthy for the baby" and a 5 for item 40 "Using a feeding method that is healthy for my baby is...". These scores are multiplied for an item attitudinal score of 10.

1. Sum all the multiplied scores for “Positive exclusive Breastfeeding Beliefs” scores.
2. The higher the score, the greater the positive exclusive breastfeeding beliefs.
3. Normative Beliefs (NorB) Scale- corresponding to social support

a. Multiply each belief statement by its corresponding motivation to comply statement. The items to be multiplied are as follows: 26,61; 27,62; 28,63; 29,64; 30,65; 31,66; 32,67; 33,68; 34,69; 35,70.

a. Sum all multiplied scores for the "Normative Beliefs" scale.

b. The higher the score, the greater the social support for exclusive breastfeeding.

1. Exclusive Breastfeeding Control Beliefs (EBCB) Scale
   1. Sum scores for items 71-80.
   2. The higher the score, the greater sense of control the woman has over her ability to exclusively breastfeed.
